# Substantial Underestimation of Post-Harvest Burning Emissions in the North China Plain Revealed by Multi-Species Space Observations

**DOI:** 10.1038/srep32307

**Published:** 2016-08-31

**Authors:** T. Stavrakou, J.-F. Müller, M. Bauwens, I. De Smedt, C. Lerot, M. Van Roozendael, P.-F. Coheur, C. Clerbaux, K. F. Boersma, R. van der A, Y. Song

**Affiliations:** 1 Royal Belgian Institute for Space Aeronomy, Avenue Circulaire 3, 1180, Brussels, Belgium; 2 Spectroscopie de l’Atmosphère, Service de Chimie Quantique et Photophysique, Université Libre de Bruxelles (ULB), 1050, Belgium; 3 LATMOS/IPSL, UPMC Univ. Paris 06 Sorbonne Universités, UVSQ, CNRS, Paris, France; 4 Royal Netherlands Meteorological Institute, De Bilt, The Netherlands; 5 Wageningen University, Meteorology and Air Quality department, Wageningen, The Netherlands; 6 Department of Environmental Science, Peking University, Beijing 100871, China

## Abstract

The large-scale burning of crop residues in the North China Plain (NCP), one of the most densely populated world regions, was recently recognized to cause severe air pollution and harmful health effects. A reliable quantification of the magnitude of these fires is needed to assess regional air quality. Here, we use an eight-year record (2005–2012) of formaldehyde measurements from space to constrain the emissions of volatile organic compounds (VOCs) in this region. Using inverse modelling, we derive that satellite-based post-harvest burning fluxes are, on average, at least a factor of 2 higher than state-of-the-art bottom-up statistical estimates, although with significant interannual variability. Crop burning is calculated to cause important increases in surface ozone (+7%) and fine aerosol concentrations (+18%) in the North China Plain in June. The impact of crop fires is also found in satellite observations of other species, glyoxal, nitrogen dioxide and methanol, and we show that those measurements validate the magnitude of the top-down fluxes. Our study indicates that the top-down crop burning fluxes of VOCs in June exceed by almost a factor of 2 the combined emissions from other anthropogenic activities in this region, underscoring the need for targeted actions towards changes in agricultural management practices.

The North China Plain covers an area of approximately 400,000 km^2^, extending over the provinces of Hebei, Shandong, Henan, Anhui, and Jiangsu. Thanks to its fertile soil, this region is one of the largest agricultural plains, and among the most densely populated areas of the world, home to over 300 million people. Wheat and maize are the two main crops in this area and their growth in rotation is a common practice in response to the increasing food demand^1^,^2^. The winter wheat growing season, between February and mid-May, is followed by harvesting in early June, whereas maize grows in July-August and is harvested in September until mid-October^3^. Large-scale agricultural burning during post-harvest season is a well-established practice which is repeated every year^3^. It helps field clearing and preparation for the next crop but has been recognized to cause extremely poor air quality and raises concerns about adverse effects on human health^4^. Straw burning limitations are actively debated in China but their implementation often faces the reluctance of farmers who use ash as a natural fertiliser. Quantifying the magnitude of the associated emissions and their impact on regional air quality is therefore urgently needed.

Bottom-up inventories based exclusively on satellite fire products generally underestimate the agricultural fires because of their small burnt area and intermittency. As an example, the VOC emission in the NCP (defined here as the region bounded by 32–40°N and 112.5–120°E) is estimated at ca. 40 Gg in June according to GFED3^5^, a factor of 10 lower than a detailed bottom-up estimate based on province-level statistical data and satellite fire products for spatial allocation^6^. Statistical information is however not available for every year, and cannot be used to assess interannual variability or evaluate emission abatement measures. In addition, the bottom-up estimation based on such statistical information for this type of emission source carries large uncertainties.

Space-based remote sensing observations of atmospheric gases offer an alternative and independent way to constrain the crop fire fluxes. Formaldehyde (HCHO) columns retrieved from the GOME satellite measurements over 1996–2001 show a large systematic column enhancement in the NCP in June, spatially coinciding with the extent of fire activity in the region, suggesting that the bottom-up biomass burning estimates are strongly underestimated in this region^7^. Latest generation satellite sounders with high signal-to-noise ratio and daily global coverage, such as the Ozone Monitoring Instrument (OMI), launched aboard the Aura platform in 2004^8^, as well as recent advances in trace gas retrievals^9^,^10^,^11^,^12^ offer the capability to derive satellite-constrained estimates of post-harvest burning emissions, to assess their year-to-year variability and to investigate their importance on regional air quality. To reach this objective, we use vertical columns of HCHO retrieved by OMI^10^ for all years between 2005 and 2012 to constrain the monthly emissions of HCHO precursors from fire burning, anthropogenic activities and vegetation. The emissions are optimised by inverse modelling of satellite HCHO columns performed using the adjoint of the IMAGESv2 chemistry-transport model^13^,^14^,^15^ (cf. Methods). The OMI-based a posteriori post-harvest emissions are evaluated against vertical columns of HCHO retrieved from the GOME-2 satellite sensor, and are compared with independent bottom-up inventories. Finally, analysis of glyoxal and NO_2_ column observations by OMI, and methanol column observations from the Infrared Atmospheric Sounding Interferometer (IASI) confirms the detection of crop burning from space and corroborates the top-down fluxes.

## Results

### Top-down agricultural fire emissions

**Figure 1:**
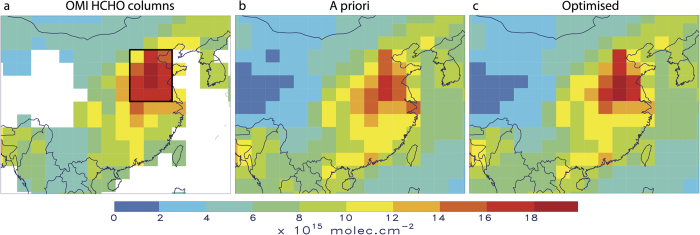
HCHO columns in June averaged over 2005–2012 (in 10^15 ^molec.cm^−2^). (**a**) Observed by OMI. (**b**) Simulated a priori model column. (**c**) A posteriori model column. The black box in (**a**) delimits the study area: 32–40°N, 112.5–120°E. The maps were generated with IDL version 8.2.3 software (http://www.exelisvis.com).

**Figure 2:**
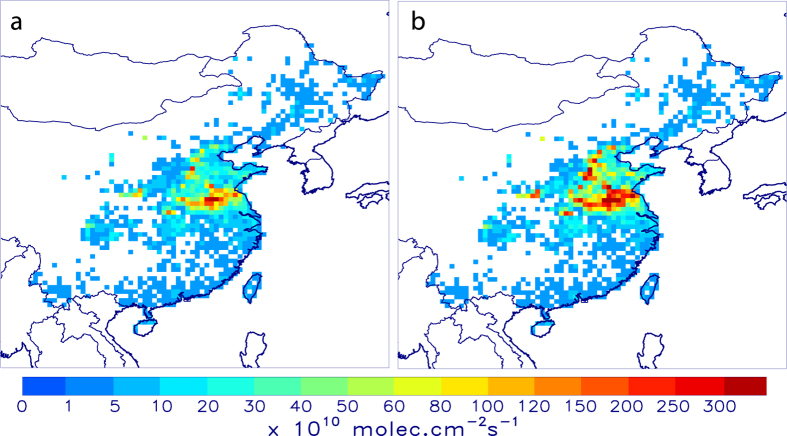
Post-harvest VOC burning fluxes in June expressed in 10^10 ^molec.cm^−2^ s^−1^. (**a**) As estimated by the a priori inventory^6^. (**b**) Average over 2005–2012 constrained by the OMI observations. The maps were generated with IDL version 8.2.3 software (http://www.exelisvis.com).

**Figure 3:**
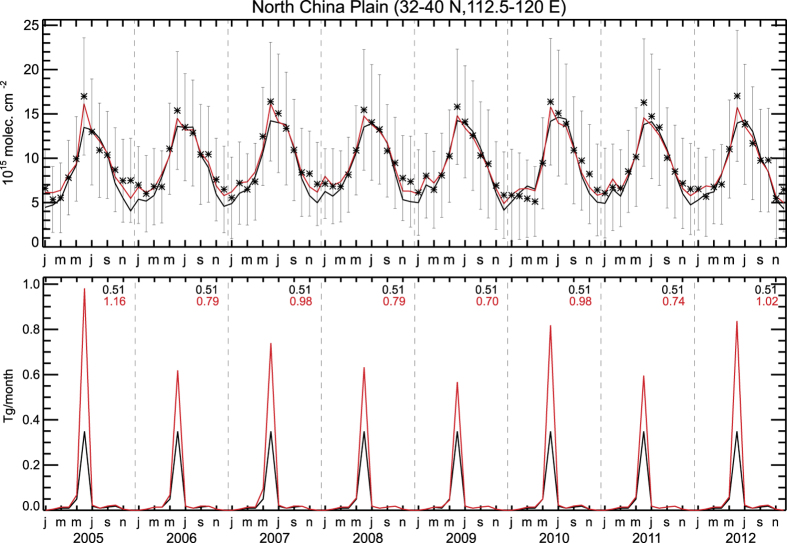
Monthly HCHO columns and agricultural burning fluxes between 2005 and 2012 in the North China Plain. (**a**) Time series observed by OMI (black asterisks and error bars), a priori model (black solid line), optimised (red). (**b**) A priori biomass burning emission fluxes (constant for all years, black), and optimised fluxes (red). Annual fluxes (in TgVOC) are shown inset using the same color code.

The OMI observations reveal enhanced formaldehyde columns in June in the NCP for all years of the study period, as illustrated in Figs 1, 2, 3. The observed columns are higher in June than in the adjacent months, by about 50% and 15% compared to May and July, respectively, pointing to the significant source from agricultural burning alongside the anthropogenic and biogenic sources of this region. The seasonal cycle of the observed HCHO columns exhibits a large amplitude with a minimum in winter (6–7 × 10^15 ^molec.cm^−2^) and maximum in summer (12–17 × 10^15^ molec.cm^−2^), and in particular in June (15–17 × 10^15 ^molec.cm^−2^). This variability is mostly due to the higher photochemical activity in summertime, as the biogenic isoprene emissions are shown to be very small for croplands^16^. In winter, formaldehyde production from anthropogenic hydrocarbon oxidation is diluted, due to slower oxidation rates associated to low solar radiation.

The a priori model simulation reproduces well the seasonal cycle of formaldehyde (Fig. 3(a)), except in winter and in June, when the model persistently underestimates the observations by about 30% and 16%, respectively.

The optimisation setup adopted to infer VOC fluxes minimising the model-observation mismatch, uses satellite columns observed over a complete year. The top-down fluxes from all emission categories are derived simultaneously. Although the emission inversions were conducted at 2° × 2.5° resolution, we also used a regional version of the same model running at 0.5° × 0.5° to verify that resolution has only a limited impact on average modelled HCHO columns in this region, in spite of the non-linearity in tropospheric chemistry and the strong spatial heterogeneity of emissions (Fig. S1).

The optimisation leads to a substantial increase of the agricultural waste burning source in June, by a factor of 2 on average for 2005–2012 with respect to the a priori inventory^6^. The top-down mean annual flux is estimated at 900 Gg, i.e. 76% higher than the a priori estimate (510 GgVOC, Fig. 3(b)). This increase improves considerably the agreement with the observations in June (Fig. 3(a)), reducing the mean discrepancy from 16% in the a priori to 6% in the a posteriori (OMI: 16.2 × 10^15 ^molec.cm^−2^, a priori model: 13.9 × 10^15 ^molec.cm^−2^, top-down: 15.3 × 10^15 ^molec.cm^−2^), although for some years the optimised columns remain slightly underpredicted after optimisation. The spatial distribution of the optimised biomass burning fluxes (Fig. 2) suggests the strongest enhancements (factor of 3) at the northernmost latitudes (>36°N) and substantial increases (factor of 2.5) between 34°N and 36°N. Moderate emission increases (by 70%) are derived in the southern part of the NCP, which is the dominant emitting region^6^ (Table S1).

In order to provide a better match with OMI observations (Fig. 3(a)), anthropogenic VOC emissions are increased during the winter months by about 25% on average for all years, whereas in summertime, the observations suggest a posterior decrease of the anthropogenic source, by about 10% in comparison to the a priori inventory (REASv2) in June (Fig. S2). The optimised anthropogenic fluxes during summer, approx. 470 Gg/month, are in excellent agreement with the independent flux estimate of 460 Gg/month from the Multi-Resolution Emission Inventory for China (MEIC)^17^. Contrary to earlier knowledge, the crop fire burning source of VOCs in the NCP in June outweighs the combined emissions from all other human-related activities (power generation, industrial/residential sector, transportation) by about a factor of two.

The errors on the a posteriori emissions are calculated by applying an off-line iterative approximation of the inverse Hessian matrix, as described in Methods. The error on the total pyrogenic NMVOC emissions in the NCP in June is lowered from a factor of 3.7 in the a priori to a factor of 1.8 after optimisation. For anthropogenic emissions, the reduction is considerably less important, from a factor of 1.45 (a priori) to a factor of 1.37 (optimised).

### Impact on the atmospheric composition

Post-harvest burning in June is estimated to have released on average 735 GgVOC (Table S1), as well as 2.1 TgCO, 25 GgNOx-N, and 0.1 TgC of carbonaceous aerosols, based on emission factor estimates for agricultural waste burning^18^. Note that the figure for NOx would be doubled if the emissions factor estimate from a recent compilation of measurements performed in fresh smoke^19^ were used;in any case, this contribution remains minor in comparison with the anthropogenic emission fluxes in the NCP in June (180 GgNOx-N^20^).

To quantify the impact of these emissions on air pollution, we perform simulations for a specific year (2010) using the model in regional mode at 0.5° resolution (Fig. S3). Compared to a model simulation ignoring those emissions, the average near-surface ozone in the NCP is increased by 7%, with the largest increases (up to 20%) calculated in the south-central part of the region. The dominant cause of this increase is the large contribution of crop fire emissions, 65% and 32%, to the total emissions of VOC and CO in the NCP, respectively. The observed seasonal variation of ozone in the NCP shows a clear maximum in June^21^,^22^,^23^, in agreement with the model. Meteorological factors are believed to be its primary cause, with however a significant contribution of crop residue burning. The calculated values in June agree fairly well with observations e.g. at Mount Tai^21^, with 85 ppbv observed vs. 70 ppbv in the model, and at Miyun^22^ (71 vs. 73 ppbv). Observations of lower tropospheric ozone by IASI^23^ show a similar pattern, with 26–30 Dobson Units (DU) below 6 km altitude in June (32 DU in the model), and lower values in May and July (28–29 DU in the model).

The calculated impact on near-surface CO and HCHO is important, +25% and +50% on average, respectively. The enhanced emissions lead to an overall decline of OH levels, between 5 and 25% within the boundary layer whereas HO_2_ levels increase by 10–18%, mainly due to conversion of OH to HO_2_ upon reaction with CO and VOC. The calculated impact of agricultural fires on NOx concentrations is low (+1% on average), owing to the low NOx emission factors for agricultural fires^18^,^19^. Finally, in the presence of crop fires, the fine aerosol loadings are increased by about 18% or ca. 10 *μ*g m^−3^ compared to the values estimated without crop burning (30–60 *μ*g m^−3^). The calculated concentrations lie within the wide range of measured PM_2.5_ levels during spring in the NCP, e.g. 25 *μ*g m^−3^ in Xianghe^24^ and 177 *μ*g m^−3^ in Nankou^25^.

### Interannual emission variability

The interannual variability of the observed columns in June is moderate with ca. 10% difference between the highest (2012) and lowest years (2006 and 2008) of the studied period (Fig. S4). The derived flux estimates, however, exhibit a much stronger interannual variability, the difference between the maximum and minimum top-down emission in June reaching ca. 50–70%. This apparent discrepancy is largely explained by the important contribution of other HCHO sources to the total column, including local anthropogenic emissions (ca. 17%), isoprene (5%) and more importantly, the background production of HCHO due to oxidation of methane and other VOCs imported to the NCP from neighbouring regions, which we estimate to about 50% based on a sensitivity calculation. The strongest flux enhancements are found in 2005, 2010, and 2012, with increases from 350 GgVOC in the bottom-up inventory to 996, 831 and 850 GgVOC, respectively, while in 2008, 2009 and 2011 the emission increased by less than a factor of 2 compared to the a priori (Table S1).

The lower crop burning fluxes in 2008 and 2009 are likely due to regulation measures aimed to ensure cleaner air conditions during the 2008 Beijing Olympics. Open field burning was banned in Northern China from May to September 2008^26^, as demonstrated by the smaller number of MODIS fire counts (−60%) in 2008 compared to 2007. The fire counts increased again gradually after 2009, and a maximum was observed in 2012 (Fig. S5). The lower observed HCHO columns and fire counts in June 2011 compared to 2010 and 2012 might be attributed to reduced crop yields due to a major drought event, not unusual in this region^27^. Indeed, ERA-Interim analyses^28^ show that the region received about twice lower rainfall beween January and May in 2011 compared to other studied years, which likely caused decreased crop yields and agricultural waste.

The top-down emissions, expressed as burnt biomass (in TgC), are compared with four independent inventories for biomass burning in Fig. S6. Two versions of the Global Fire Emissions Database are used, version 3^5^ and the GFED4s which includes burned area from small fires based on active fire detections^29^; the Global Fire Assimilation System (GFAS) based on assimilation of fire radiative power observed from MODIS aboard the Terra and Aqua satellites^30^, and the Fire Inventory from NCAR (FINN) version 1.5, an updated version of the FINN inventory^31^. On average over the eight years the top-down emissions are about 14, 7 and 4 times larger than in GFAS, GFED4s, and FINN, respectively, whereas the GFED3 emissions are even lower. In terms of interannual variability, the GFED4s and GFAS fluxes correlate better with the satellite-derived fluxes (*r* = 0.58) than the FINN emissions (*r* = 0.45). Finally, the updated fluxes over NCP in June are found to be well correlated spatially with the MODIS fire counts (*r* > 0.7 for most years). Their interannual variability is also very similar (Fig. S5), especially between 2006 and 2012 (*r* = 0.73).

### Sensitivity inversions

The uncertainty on the top-down source is evaluated by conducting sensitivity optimisations to quantify the role of key parameteters, namely (i) the assigned a priori flux errors, (ii) the fire-related emission factors, (iii) the inversion setup, and (iv) the diurnal cycle of fire activity. Although this evaluation does not cover all possible sources of errors, it accounts for the most important uncertainties associated with the model and the inversion framework.

As not only the a priori emissions themselves but also their uncertainties are difficult to evaluate, the assigned errors on the a priori biomass burning emissions were doubled and halved in DE and HE optimisations (Table 1), respectively, with respect to the initially assigned error (cf. Methods). As expected, the DE inversion realises a stronger bias reduction, compared to the a priori, 70% vs. 56% in the standard (STD) optimisation. The better agreement with the observations is realised by even larger emissions, 950 Gg in DE vs. 850 Gg and 690 Gg in STD and HE, respectively. The increase is especially pronounced in the northernmost part of the NCP (ca. factor of 5), apparently reflecting a misrepresentation of the spatial patterns of the a priori emissions, for reasons currently unclear (Table 1, Fig. S7).

Another important source of uncertainty lies in the assumed biomass burning emission factors. The standard setup employs the widely used updated (2011) emission factors from ref. 18, whereas a recent compilation of emission factors based on measurements performed in fresh smoke^19^ is adopted in two sensitivity inversions, AK and AKU. The agricultural waste emission factors are similar in both databases for most compounds, however large differences exist for individual compounds, e.g. acetaldehyde (2.77 g/kg in ref. 18, 1.24 in ref. 19), glyoxal and methylglyoxal (1.0 and 0.73 g/kg in ref. 18, 0 in ref. 19). Comparison with measurements of crop burning emission factors in China^32^ indicates that the emission factors adopted in our standard simulation^18^ are likely too high for oxygenated compounds (as they are not corrected for chemical production in the fire plumes) and possibly too low for pure hydrocarbons such as alkanes, alkenes and aromatics. A large source of variability and uncertainty might lie in the complex influence of fuel moisture on the emission factors^33^. Overall, the a priori biomass burning emission in June is by 20% lower when the newer datasets^19^,^32^ are used (280 vs. 350 Gg, Table 1). The optimisation AKU, also based on ref. 19, acknowledges the fact that a significant mass fraction of the gas-phase VOC emitted by biomass burning is unidentified, as revealed by field and laboratory measurements^34^,^35^. Therefore, AKU includes an additional non-methane VOC source in the model, of magnitude equal to the known source^19^, distributed as 50% propene and 50% monoterpene, in such a way that the resulting mix has a HCHO mass yield similar to the overall mass yield of the known source. Due to their short lifetimes, these VOCs are oxidised closer to their emission source than the more long-lived identified VOCs. As a result, the VOC mix in AKU leads to higher HCHO levels directly above the emitting region compared to AK and the standard STD case. After optimisation, both AK and AKU inversions result in a similar a posteriori flux (ca. 750 Gg), yet a better agreement with the measurements is achieved in the AKU optimisation, i.e. significant reduction of the bias (Table 1, Fig. S7), due to the shorter lifetime and higher a priori emissions of HCHO precursors in this scenario. Note that in this case, the increase relative to the a priori is moderate (40%).

To evaluate the role of interferences associated with the optimisation of non-fire emissions, we perform an optimisation varying only the biomass burning source, while the anthropogenic and biogenic VOC source remain equal to the a priori (BB inversion, Table 1). This setup worsens the agreement with the observations (Table 1), whereas the inferred flux is by 20% lower (ca. 650 Gg) than for the standard inversion (850 Gg). Finally, to account for the uncertainty associated with the choice of the diurnal cycle profile for fire emissions, and since MODIS fire counts indicate that the diurnal cycle of fire activity over large croplands of central Eurasia is insignificant^36^, we performed the optimisation NDC (no diurnal cycle) assuming no diurnal variation in agricultural fires. The resulting bias and root mean square deviation are very similar in comparison with the standard inversion and a slightly higher (+3%) a posteriori flux is estimated. Overall, the top-down emission estimates appear robust across the different sensitivity cases, and indicate a flux increment of at least a factor of two compared to the a priori inventory.

## Discussion

**Figure 4:**
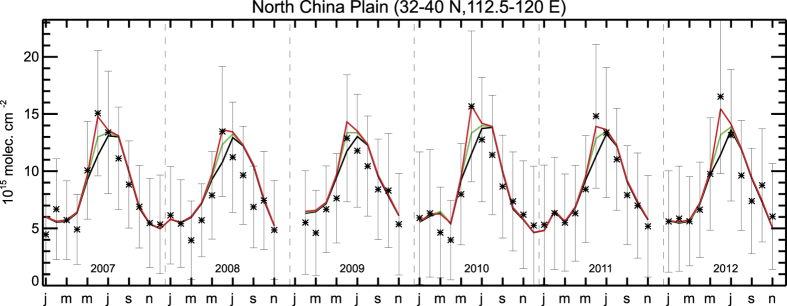
Time series of monthly HCHO columns observed by GOME-2 (black asterisks and error bars) and from forward simulations neglecting crop fire fluxes (black), obtained from the a priori inventory^6^ (green), and using the OMI-based crop fire estimates (red).

The strong increase in the satellite-derived fluxes is further corroborated by satellite observations of HCHO retrieved by the GOME-2 sensor onboard Metop-A which measures HCHO columns daily with a morning overpass time (9:30 local time). As in the case of OMI (afternoon overpass 13:30), the GOME-2 dataset indicates a pronounced maximum in the NCP in June, by ca. 10–50% higher than the preceding and following months. The GOME-2 peak is by about 10% lower than the corresponding maximum observed by OMI (Figs 3 and 4), the difference being primarily due to the diurnal cycle of HCHO columns, which is mostly affected by the diurnal cycle of emissions, as well as by OH and solar radiation levels^15^. The current version of the GOME-2 data is briefly described in Section 1 of the Supplement and in detail in ref. 10. As seen in Fig. 4 (Fig. S8), the GOME-2 columns strongly support the findings inferred from OMI observations. The initial model underprediction by about 10–15% in June disappears almost completely when the OMI-based emissions are used, except to some extent in 2011 and 2012, where the model remains too low, as was already the case with respect to OMI. A sensitivity inversion using GOME-2 observations as top-down constraints for 2012 leads to an a posteriori flux of 968 GgVOC, i.e. 14% higher than the estimated emissions using OMI observations (Table 1). The mean bias between the GOME-2 columns and the optimised model results (2.54 × 10^14^ molec.cm^−2^) is about factor of 2 lower than in the a priori simulation, and the a posteriori root mean square deviation is reduced by 30%.

**Figure 5:**
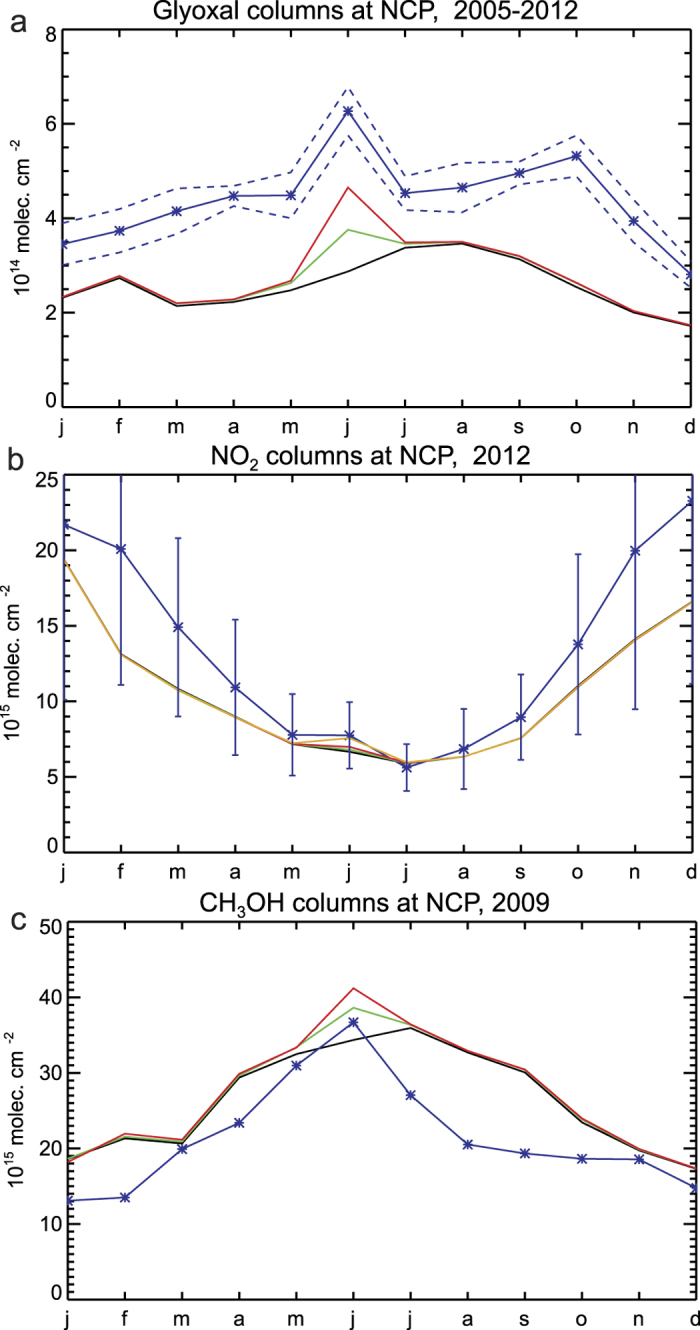
Seasonal variability of (**a**) glyoxal, (**b**) NO_2_, and (**c**) CH_3_OH columns observed and modelled over the North China Plain. Observations are shown in blue. Model results using crop fire fluxes set to zero, obtained from the a priori inventory^6^, and using the OMI-based estimates derived in this study are shown in black, green and red, respectively. The dashed lines in (**a**) denote the standard deviation of the mean OMI glyoxal column over 2005–2012. The error bars in (**b**) represent the retrieval error of the DOMINOv2 dataset for the year 2012. The model NO_2_ column obtained using OMI-based fluxes and emission factors for agricultural waste burning from ref. 19 are shown in orange.

The effects of crop residue burning in the NCP are detected from space in the observations of glyoxal (CHOCHO) retrieved by the OMI instrument. Glyoxal is formed in the oxidation of VOCs released by the biosphere, fires and human activities. The total glyoxal source is believed to be predominantly secondary and biogenic in origin^37^,^38^. Current models, however, largely underestimate the observed glyoxal satellite columns suggesting the existence of, as yet unknown, additional sources^37^. To our best knowledge, only 18% of the total glyoxal budget is due to biomass burning (primary and secondary), but this contribution is more pronounced at regional scale, where it often prevails over other sources^38^. The monthly OMI glyoxal columns over the study region averaged over 2005–2012 are shown in Fig. 5a (Fig. S9). The retrieval algorithm, initially developed for GOME-2 observations^12^, was updated to account for the specifics of OMI (cf. Supplement, Section 2). The 2005–2012 June mean observed glyoxal column in the NCP amounts to 6.3 × 10^14 ^molec.cm^−2^, higher by 1.8 × 10^14 ^molec.cm^−2^ than the mean value of May and July. A similar enhancement (1.5 × 10^14 ^molec.cm^−2^) is derived by the model when using the OMI-based optimised fluxes, whereas the a priori simulation strongly underestimated the peak (0.7 × 10^14 ^molec.cm^−2^, Fig. 5a). Therefore, despite the model underprediction over the study region pointing to missing glyoxal sources present all year long, the column enhancement due to post-harvest burning is well reproduced by the model. This result lends confidence to the emission speciation profile, and in particular the glyoxal emission factor for agricultural fires (1.12 g/kg dry matter^18^), although this production might be largely due to secondary formation due to fast oxidation of very short-lived, mostly unknown precursors. The secondary glyoxal peak observed in October (Fig. 5a), not reproduced by the a posteriori model, might be partly due to fires set up for clearing fields after the maize harvest season. These fires cause a secondary peak in the seasonal profile of the a priori emissions^6^, but of very weak amplitude, about a factor of 20 lower than the June peak. Furthermore, the OMI HCHO dataset does not show a consistent pattern, with a local maximum in October seen only for two years (2006 and 2012) out of 8. Note that the higher zenith angles and lower magnitude of HCHO columns reduce the signal-to-noise ratio and the ability of the inversion to retrieve reliable fluxes in October.

The higher top-down emissions lead to a better agreement with satellite obeservations of NO_2_ from the OMI instrument (DOMINOv2^11^). The strong seasonal variation in the observed columns (Fig. 5b) is due to changes in the chemical lifetime of NOx and is relatively well reproduced by the model. Since anthropogenic emissions are not expected to vary much between May and July, the observed column enhancement in June, relative to May and July is to a large extent due to pyrogenic emissions. Although relatively small, this enhancement (1.05 × 10^15 ^molec.cm^−2^) is higher than predicted by the optimised model (0.27 × 10^15 ^molec.cm^−2^, Fig. 5b). This likely points to an underestimated NO_2_ emission factor for agricultural waste used in the model, 2.44 g per kg of dry matter. Indeed, adopting the higher value (4.769 g/kg) suggested by^19^ and corroborated by an independent study based on OMI NO_2_ measurements^39^, leads to a modelled enhancement of 0.97 × 10^15 ^molec.cm^−2^, in very good agreement with the observations.

Additional evidence for the intense burning in the NCP is given by methanol columns retrieved from the thermal infrared sensor IASI^40^ (cf. Supplement). Methanol is directly emitted by vegetation, anthropogenic activities, and open fires (55%, 5% and 3% of the global budget, respectively), whereas the remainder is due to oceanic emissions and secondary production^41^. In Fig. 5c the observed monthly averaged methanol columns in 2009 over the NCP are compared with the model. The model overestimates the observations between June and September (Fig. S9), likely due to an overestimation of biogenic emissions. However, use of the optimised emissions leads to a column enhancement in June of 6.3 × 10^15 ^molec.cm^−2^, very close to the observed enhancement (7.7 × 10^15 ^molec.cm^−2^). Note that for methanol, the emission factors for agricultural burning are similar across recent evaluations^18^,^19^,^32^ (between 2.94 and 3.45 g/kg, respectively).

Finally, it should be acknowledged that a significant source of error resides in the effects of aerosols in the UV-visible retrievals for individual observations above fire scenes^7^,^42^. This impact was, however, found to be limited for monthly mean NO_2_ columns, suggesting that the effect of aerosols is reduced by spatiotemporal averaging^42^, whereas accounting for an implicit or explicit aerosol correction leads to only a 6% difference in the retrieved NO_2_ columns^43^. For fire scenes over Amazonia the impact of the aerosol correction was found to only moderately affect the HCHO columns (3%), especially when aerosols are located throughout the boundary layer^44^, as is the case for crop residue burning. Furthermore, for weak absorbers like HCHO and CHOCHO, errors on the vertical columns are as much due to slant column errors (calibration uncertainties, interferences with other species, etc.) as to errors in airmass factors.

## Methods

### Modelling

The emission inversions are performed with the IMAGESv2 global chemistry transport model for all years between 2005 and 2012 following a four-month spin-up time. The model resolution is 2° × 2.5° and is extended vertically from the surface to the lower stratosphere through 40 unevenly spaced hybrid (*σ*-p) levels. The IMAGESv2 model is also run in regional mode, at 0.5° × 0.5° resolution, in a domain delimited as 17–54°N and 73–150°E. The regional model uses boundary conditions at its lateral borders from simulations using the global model. The chemical scheme, emission databases and parameterizations of the regional model and global model are identical.

The a priori anthropogenic fluxes in IMAGESv2 are obtained from the REASv2 inventory for Asia until 2008^20^ and are set to the 2008 value beyond that year. Agricultural fire burning in China is obtained from the inventory of ref. 6 for 2006, while biomass burning elsewhere is taken from GFED3^5^ until 2011, and a climatological mean (1997–2011) for 2012. The diurnal profile for fire emissions, based on observations of a geostationary satellite^45^, peaks at ca. 13:30, while it is negligible at night^15^. Biogenic emissions of isoprene are obtained from the MEGAN-MOHYCAN-v2 global inventory driven by the ECMWF ERA-Interim meteorological fields^46^. The 2005–2012 mean annual isoprene flux in China is estimated at 7.2 Tg, with the largest emission calculated in 2007 (7.6 Tg) and the lowest in 2012 (6.8 Tg).

The HCHO simulation is thoroughly described in refs 14,15. In the current version of the model, HCHO is formed in the oxidation of 24 explicit NMVOC precursors, namely, C_2_H_2_, C_2_H_4_, C_2_H_6_, C_3_H_6_, C_3_H_8_, isoprene, *α*-pinene, HCHO, CH_3_CHO, glycolaldehyde, hydroxyacetone, methacrolein, methyl vinyl ketone, glyoxal, methylglyoxal, methanol, formic acid, acetone, methyl ethyl ketone, biacetyl, acetic acid, benzene, toluene, and xylenes. Higher hydrocarbons are represented as a lumped compound with oxidation chemistry detailed in ref. 15. The speciation profile for pyrogenic emissions follows ref. 18 with 2011 updates. The average molecular weight of the mix of pyrogenic NMVOCs is equal to 46.7 kg kmol^−1^. The chemical mechanism uses molar yields of glyoxal from benzene, toluene and xylenes of 75%, 70% and 36%, respectively^47^.

### Satellite formaldehyde columns

OMI (Ozone Monitoring Instrument), part of AURA’s payload launched in 2004, is a nadir-viewing UV-Visible sensor^8^ measuring global solar radiances backscattered from the Earth’s atmosphere daily in the early afternoon (ca. 13 h30 local time) at high spatial resolution (13 × 24 km^2^). Slant column densities of HCHO are fitted in the 328.5–346 nm spectral window based on the algorithm described in ref. 9, which has been improved (i) to reduce the O_2_-O_2_ absorption effects and minimise the interferences with other molecules, (ii) to remove spike residuals, and (iii) to reduce offsets between rows by applying a destriping correction in the background reference sector^10^. These modifications eliminate inconsistencies and unphysical results, increase the signal-to-noise ratio, and offer a higher temporal stability to the long-term dataset of OMI HCHO columns.

Monthly mean HCHO columns between 2005 and 2012 are used as constraints in a flux inversion scheme based on the discrete adjoint model of the IMAGESv2 global CTM (see below). The target region is Eastern China, and in particular the NCP delimited as 32–40°N, 112.5–120°E. Only observed columns with cloud fraction lower than 40% are used in the inversion. The error of the satellite columns is defined as the geometric mean of the retrieval error and an additional error of 2 × 10^15 ^molec.cm^−2^. Error corelations between observations are not considered. In the target region, the errors amounts to 45–48% in summertime, and up to 85% in winter. Monthly averaged columns with errors larger than 100% are not accounted for in the inversion. The model columns are sampled at the location and time of the observations. Column observations over oceans are excluded from the inversion analysis.

### Inversion of surface fluxes

This approach consists in minimising a scalar function (cost function) quantifying the mismatch between modelled and observed HCHO columns by adjusting the flux rates of the emitting sources on a monthly basis for every model grid^13^,^14^,^15^. The inversion is realised through the discrete adjoint method, i.e. a line-by-line transpose of the tangent linear model in the reverse order, which consists in calculating the partial derivatives of the cost function


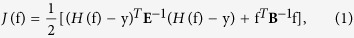


with respect to emission parameters *f* to be optimised, where *H*(f) is the chemistry-transport model operating on the control variables, *y* is the observation operator, and **E**, and **B** are the covariance matrices of the errors on the observations and the emission parameters *f*, respectively, and ^*T*^ denotes the transpose of the vector. This framework has been applied for inverting the fluxes of several atmospheric gases^13^,^48^,^49^, and is particularly appropriate for reactive compounds, as it can account for chemical feedbacks. A priori emission distributions for anthropogenic, pyrogenic and biogenic fluxes obtained from the latest available inventories are used in the model and are updated based on the satellite columns of formaldehyde through an iterative minimisation algorithm using the forward and its backward in time (adjoint) model. Of the order of 4 × 10^14^ emission parameters (of which 350 in NCP) from all categories are optimised annually for every month and per grid cell with non-zero initial emission, and the minimum is achieved once the convergence criterion, set as a reduction of the norm of the cost function gradient by a factor of 10^3^, is satisfied. The number of iterations required to achieve convergence varies from year to year depending on the availability and errors of top-down constraints and generally lies between 30 and 50.

### Inversion setups

Annual inversions have been carried out between 2005 and 2012 using monthly HCHO columns, and VOC fluxes from anthropogenic, biomass burning and isoprene are updated on the global scale. The errors on the emission parameters, i.e. the diagonal elements of the matrix **B**, are assumed to be a factor of 2, 2.5, and 3 for anthropogenic, biogenic, and fire emissions, respectively. A larger error for the pyrogenic source is assumed over the NCP (factor of 7) to account for the large uncertainties and variability of this source. The off-diagonal elements of **B** represent spatiotemporal correlations between the errors of the emisssion parameters^14^,^15^. In our inversion experiments, the spatial terms are as in ref. 14, whereas the temporal terms decrease linearly from 0.2 (for successive months) to zero (for a 6-month lag) for the biogenic and pyrogenic fluxes, and from 0.92 to 0.6 for the anthropogenic fluxes. No interannual correlations are assumed. Finally, the matrix **E** is assumed diagonal, and the errors on OMI HCHO columns are defined as previously.

### Calculation of the a posteriori errors

The posterior error covariance matrix of the control variables is defined as the inverse Hessian matrix of the cost function at its minimum. Assuming that the model is linear or weakly non-linear, the a posteriori error covariance matrix is related to the Hessian of the cost function *J* through the expression^13^:


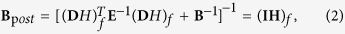


where 
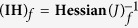
 is the inverse Hessian matrix calculated at the point *f*, **D***H* is the Jacobian matrix of the model, and **E** and **B** are as in Eq. (1). To calculate the inverse Hessian matrix we apply the Davidon-Fletcher-Powell (DFP) iterative approximation^50^, which uses the updated information obtained at each step *k* of the minimisation:


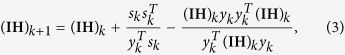


where 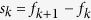, 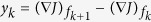, 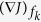 is the gradient of *J* at the point *f*_*k*_, and the initial inverse Hessian matrix is taken to be equal to **B**. The approximate inverse Hessian matrix is then obtained through recursive application of the DFP updating formula on the vectors *f*_*k*_ and 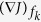 calculated after each iteration. The standard errors of the a posteriori variables *f* are the square roots of the diagonal elements of the inverse Hessian matrix, and the error reduction is defined as the ratio of the a priori to the a posteriori error.

## Additional Information

**How to cite this article**: Stavrakou, T. *et al*. Substantial Underestimation of Post-Harvest Burning Emissions in the North China Plain Revealed by Multi-Species Space Observations. *Sci. Rep.* **6**, 32307; doi: 10.1038/srep32307 (2016).

## Supplementary Material

Supplementary Information

## Figures and Tables

**Table 1:** Root mean square deviation (RMSD), root mean square relative error (RMSRE) and bias between the OMI HCHO columns and the model predictions before and after the sensitivity inversions over the study region in June 2012.

Simulation	RMSD	RMSRE %	Bias	Flux Gg
	10^15 ^molec.cm^−2^		10^15 ^molec.cm^−2^	
A priori	3.40	19.8	3.05	350
STD	2.20	13.2	1.32	850
DE	1.82	11.1	0.90	950
HE	2.75	16.2	2.05	690
AK	2.36	14.1	1.49	750
AKU	2.03	12.2	0.91	780
BB	2.29	13.6	1.59	647
NDC	2.18	13.1	1.42	878

STD: standard inversion; DE and HE: doubled or halved a prori flux errors; AK emission factors from^19^; AKU: as AK with additional source to account for unidentified VOCs; BB: only biomass burning fluxes are varied; NDC: no diurnal cycle of fires. Note that the a priori crop fire fluxes in AK, AKU inversions are equal to 280 and 560 Gg in June 2012, respectively.
