# Supplementary material for: Substantial Underestimation of Post-Harvest Burning Emissions in the North China Plain Revealed by Multi-Species Space Observations
Source: Sci Rep. 2016 Aug 31;6:32307. doi: 10.1038/srep32307 (PMC5006073; doi:10.1038/srep32307)
Supplement: Supplementary Information [file srep32307-s1.pdf]

Supplementary Material for

Substantial Underestimation of Post-Harvest Burning Emissions in the North China Plain Revealed by Multi-Species Space Observations

T. Stavrakou[1⋆], J.-F. Müller[1], M. Bauwens[1], I. De Smedt[1], C. Lerot[1], M. Van Roozendael[1], P.-F. Coheur[2], C. Clerbaux[2,3], K. F. Boersma[4,5], R. van der A[4], Y. Song[6]

[1] Royal Belgian Institute for Space Aeronomy, Avenue Circulaire 3, 1180, Brussels, Belgium

[2] Spectroscopie de l'Atmosphère, Service de Chimie Quantique et Photophysique, Université Libre de Bruxelles (ULB), 1050, Belgium

[3] LATMOS/IPSL, UPMC Univ. Paris 06 Sorbonne Universités, UVSQ, CNRS, Paris, France

[4] Royal Netherlands Meteorological Institute, De Bilt, the Netherlands

[5]Wageningen University, Meteorology and Air Quality department, Wageningen, the Netherlands

[6] Department of Environmental Science, Peking University, Beijing 100871, China

⋆ E-mail : Trissevgeni.Stavrakou@aeronomie.be

⋆ E-mail : Trissevgeni.Stavrakou@aeronomie.be

Contents

1 GOME-2 formaldehyde columns 3

2 Glyoxal columns observed by OMI 3

3 Methanol column observations from IASI 4

4 Tables 7

5 Figures 8

References 16

1 GOME-2 formaldehyde columns

Formaldehyde vertical columns have been retrieved from the GOME-2 sensor aboard the MetOp-A satellite using the BIRA-IASB algorithm (v14)[S1]. Applied consistently to both GOME-2 and OMI measurements, this algorithm is used to produce global distributions of HCHO representative of mid-morning and early afternoon conditions. Its main features include (i) a new iterative DOAS scheme involving three fitting intervals to better account for the O_2-O_2 absorption; (ii) the use of earthshine radiances averaged in the equatorial Pacific as reference spectra; (iii) a destriping correction and background normalisation resolved in the across-swath position. The air mass factor calculation[S2] is based on scattering weighting functions evaluated with the LIDORT v3.3 radiative transfer model[S3]. The a priori profile shapes are provided on a daily basis by simulations performed by the IMAGES model, at 09:30 a.m. for GOME-2. As surface reflection database, the OMI-based dataset[S4] is used. Radiative cloud effects are corrected using the independent pixel approximation[S5] and the GOME-2 O_2 A-band Frescov6 cloud product[S6].

2 Glyoxal columns observed by OMI

Glyoxal vertical columns have been retrieved from the OMI instrument using the BIRA-IASB algorithm relying on the DOAS approach. A slant column density (concentration integrated along the effective light path) is first in-

verted using the glyoxal absorption bands located between 435 and 460 nm, and then converted into a vertical column density with air mass factor (AMF) computations. The main features of the algorithm remain the same as in the original version developed for GOME-2/Metop-A[S7]. In particular, a two-step DOAS fit is carried out to take the liquid water absorption into account and limits the impact of spectral interferences with glyoxal, a normalization procedure based on the Pacific sector ensures a constant background level, and AMFs are computed using weighting functions computed with the radiative transfer model LIDORT[S3] and daily a priori glyoxal profile shapes provided by the CTM IMAGES. The database for cross sections used for the DOAS fit was updated with more recent data and now also includes a second NO_2 cross-section at room temperature[S8, S9]. The normalization procedure has been adapted to OMI and is performed separately for the 60 rows of pixels across-track in order to remove possible offsets in the retrieved slant columns intrinsic to imaging instruments. Finally, the higher spatial resolution of OMI and the resulting large number of pixels allows a more stringent filtering for the cloud contamination. Only clear sky pixels (cloud fraction less than 20%) are preserved and no cloud correction is applied.

3 Methanol column observations from IASI

IASI instrument is a nadir-viewing Fourier Transform Spectrometer instrument that measures in the thermal infrared, between 645 and 2760 cm^{-1}. It

was launched onboard the MetOp-A platform in October 2006, and a second instrument was launched in September 2012. Currently both instruments orbit the Earth, each delivering near global coverage twice daily at ca. 9:30 local time (AM and PM). A detailed description of IASI can be found in [S10]. Methanol is observable in the infrared using its strongest absorption band centered at 1033 cm^{-1}. However, its detection is challenging due to its weak absorption, hampered by interferences of other molecules in the same spectral range[S11].

The method applied to retrieve methanol columns is based on brightness-temperature differences (ΔT_b) between the channel of 1033 cm^{-1} and neighbouring channels, where the absorption is weak. The retrieval consists in three steps : ΔT_b are determined on the global scale, a correction is applied in order to minimize the impact of ozone and water vapor interferences, and ΔT_b are converted to total columns using a radiative transfer model. Radiative transfer calculations were carried out over a limited number of world regions, and the conversion factors were determined through matching the retrieved columns on the ΔT_b. These factors were then applied to deduce global total methanol columns[S11]. Only daytime (9:30 AM) cloud free scenes (cloud fraction below 2%) were considered in the retrievals. One complete year of methanol retrievals (2009) was performed based on this method. The error on the monthly column averages was estimated at about 50% over continents, but could be higher when thermal contrast is low. The IASI observations have been used as top-down constraints in an inverse modelling

study in order to derive global methanol emissions[S12].

4 Tables

Table S1: A priori and satellite-derived crop fluxes over the North China Plain in June expressed in GgVOC.

Prior	2005	2006	2007	2008	2009	2010	2011	2012	2005-2012
350	996	628	750	642	576	831	604	850	735

Table S2: A priori and updated NMVOC emission estimates (in Gg) from agricultural burning in June for the study region. The a priori refers to year 2006, and the optimised emissions are averaged over 2005-2012.

Latitude	Longitude	A priori	Optimised
	112.5-115 E	37.5	75.5
32-34 N	115-117.5 E	105.3	155.7
	117.5-120 E	39.5	71.2
	112.5-115 E	14.5	38.1
34-36 N	115-117.5 E	42.6	107.4
	117.5-120 E	42.9	90.5
	112.5-115 E	24.6	79.8
36-38 N	115-117.5 E	14.3	49.5
	117.5-120 E	7.3	19.4
	112.5-115 E	3.6	8.7
38-40 N	115-117.5 E	16.8	47.1
	117.5-120 E	1.9	4.3
32-40 N	112.5-117 E	350	747

5 Figures

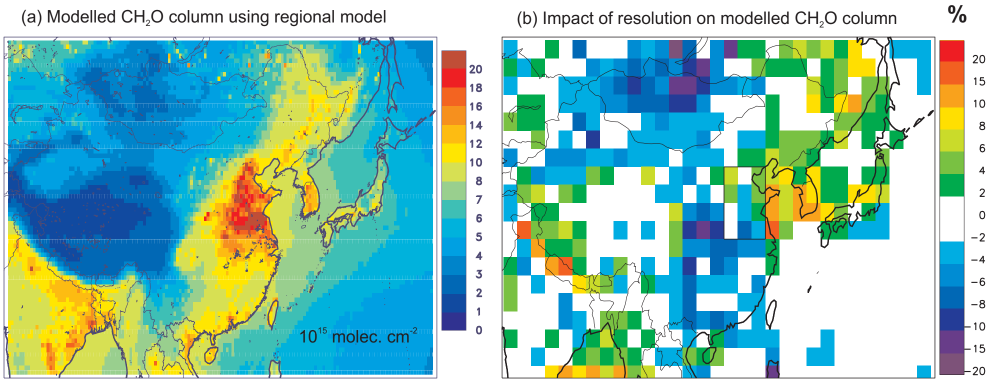

Figure S1: **Impact of spatial resolution on modelled CH_2O columns.** IMAGESv2 in regional mode ($0.5° \times 0.5°$) is used. The model is run for year 2010 with pyrogenic emissions at $0.5° \times 0.5°$ from Ref. [S13], updated using the OMI-based emission optimisation performed at $2° \times 2.5°$. **a,** Monthly averaged CH_2O columns in June 2010 (10^{15} molec.cm^{-2}). **b,** Relative percentage difference between CH_2O columns calculated with the regional model (regridded at $2° \times 2.5°$) and the global model in June 2010, using OMI-based optimised emissions. The maps were generated with IDL version 8.2.3 software (http://www.exelisvis.com).

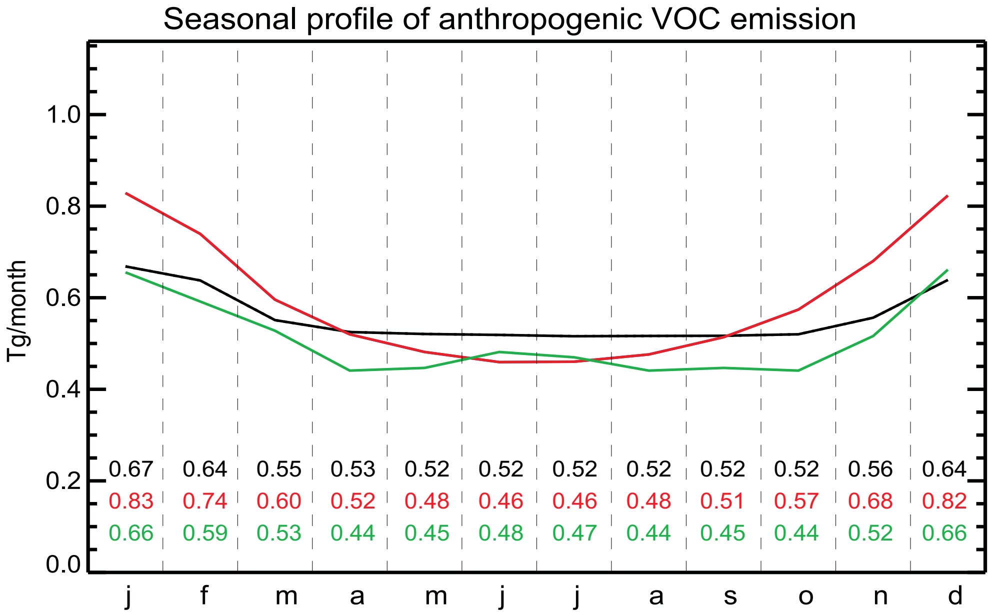

Figure S2: **Seasonal profile of anthropogenic VOC emission in the North China Plain averaged over 2005-2012.** Monthly emission estimates from the REASv2 inventory (in black) are compared with top-down estimates (red), as well as with the MEIC 2008 inventory for China (green).

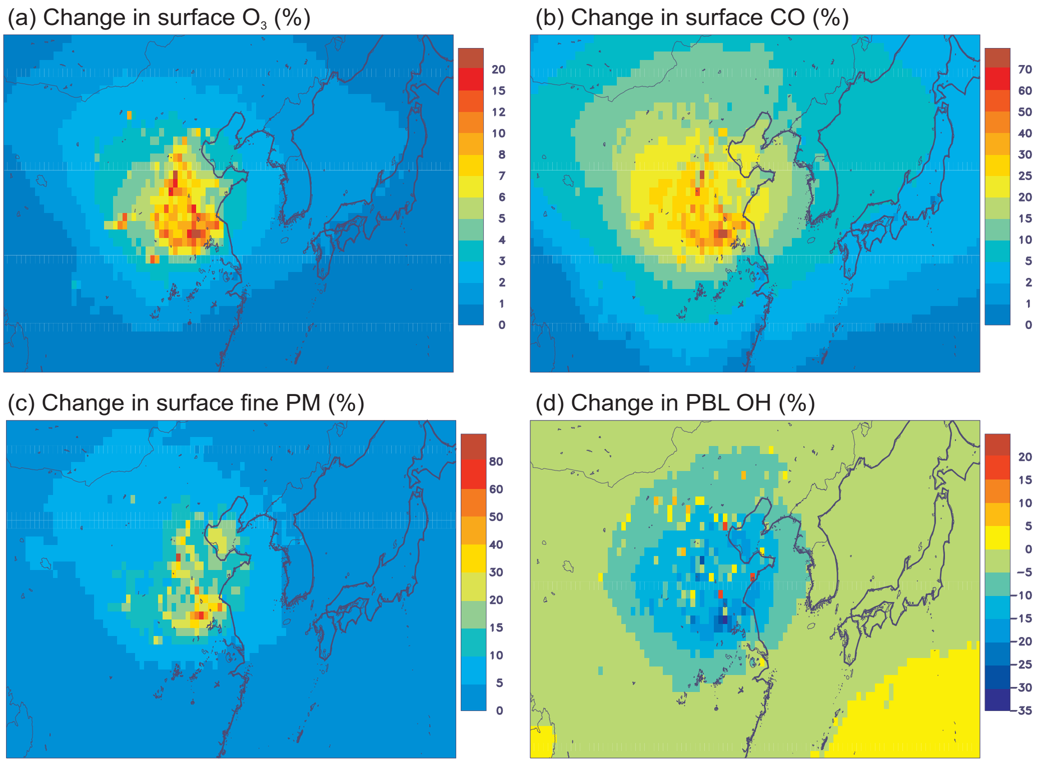

Figure S3: **Modelled changes in key chemical compounds due to agricultural fires.** The changes are calculated for June 2010 using IMAGES in a regional mode at $0.5° \times 0.5°$ resolution over East Asia (17-54°N, 73-150°E). Shown are relative changes between a simulation including pyrogenic emissions optimized in this study and a simulation ignoring those emissions. **a,** Near-surface ozone, **b,** Near-surface CO, **c,** Near-surface fine aerosol (both inorganic and organic), **d,** Average boundary layer OH. The maps were generated with IDL version 8.2.3 software (http://www.exelisvis.com).

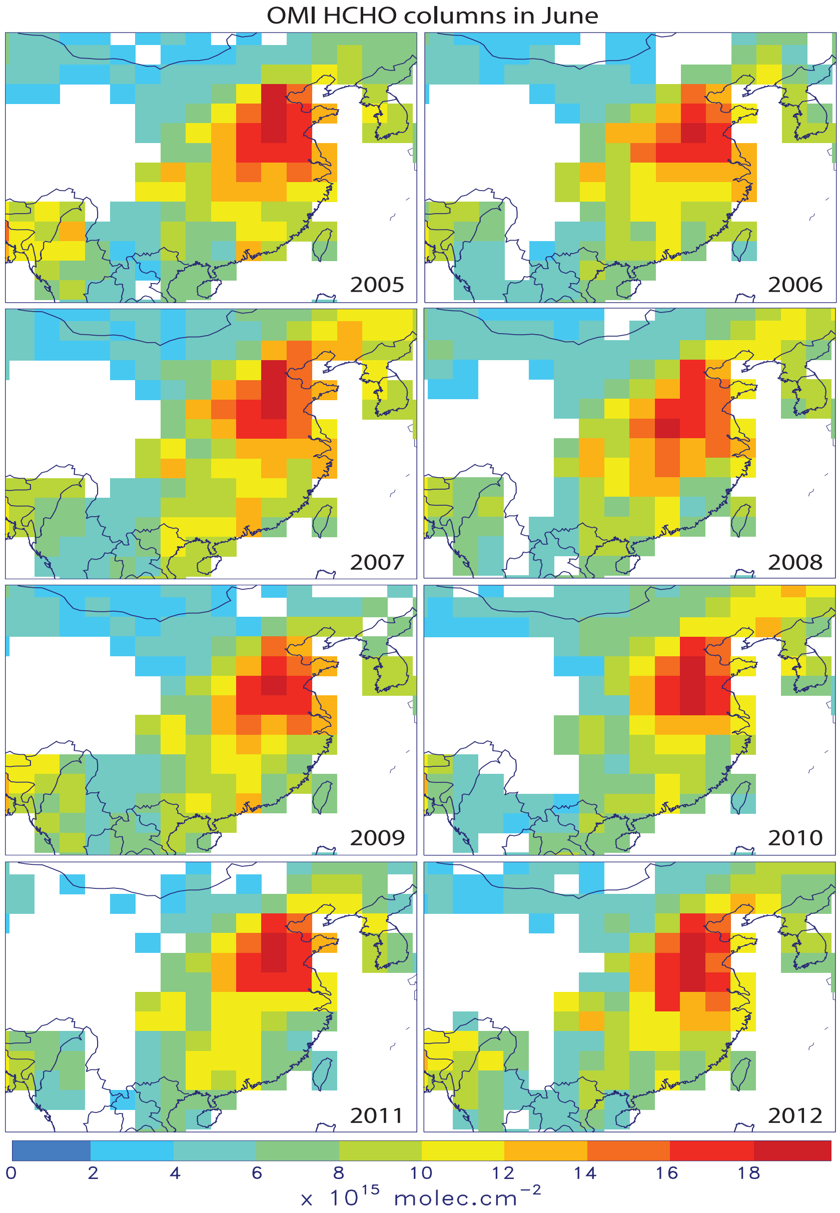

Figure S4: **Monthly HCHO columns in June observed by OMI between 2005 and 2012**. Units are 10^{15} molec.cm^{-2}. The maps were generated with IDL version 8.2.3 software (http://www.exelisvis.com).

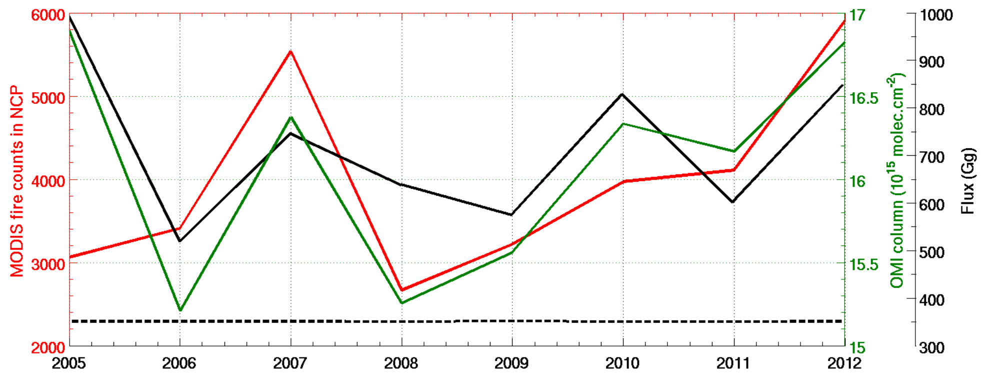

Figure S5: **Year-to-year variability (2005-2012)** of MODIS fire counts (red), OMI column of formaldehyde (green), a priori (dashed black) and OMI-based fluxes (black) over the study region in June.

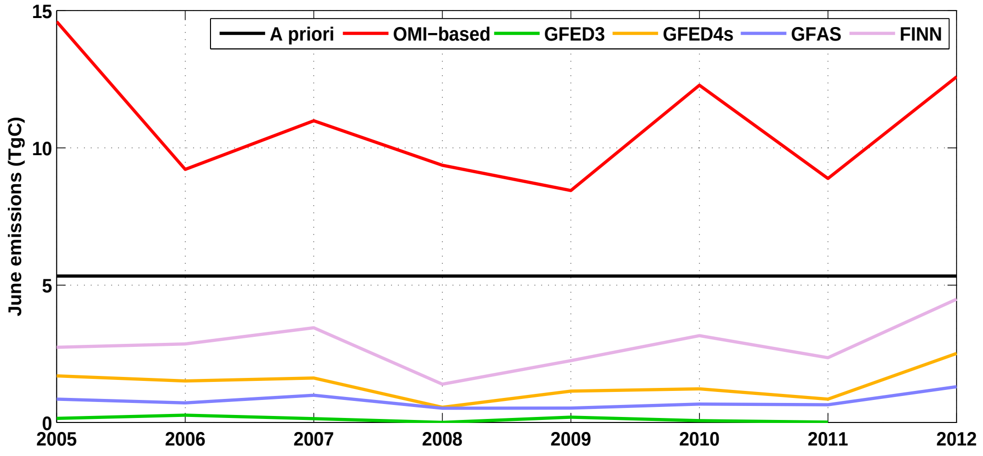

Figure S6: **Comparison between top-down biomass burning emissions (in red) in June over the study region and estimates from four bottom-up inventories** (GFED3 in green, GFED4 in orange, GFAS in blue, FINNv1.5 in pink). The a priori inventory[S13] corresponds to 2006 emissions and is used for all years (2005-2012). Units are TgC.

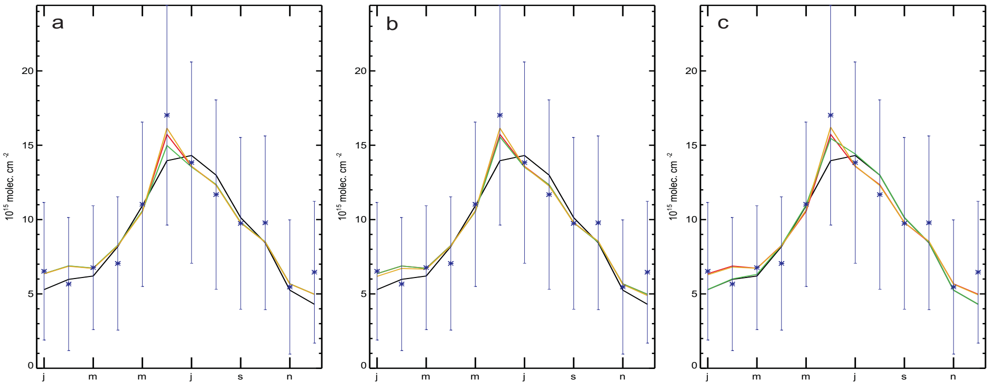

Figure S7: **Monthly HCHO columns in 2012 observed by OMI in blue), predicted by the a priori model (in black) and the optimised model (in red) over the study region (32-40 N, 112.5-120 E).** Inferred columns from the sensitivity inversions (defined in Table 1): **a,** HE in green, DE in orange, **b,** AK in green, AKU in orange, **c,** BB in green, NDC in orange.

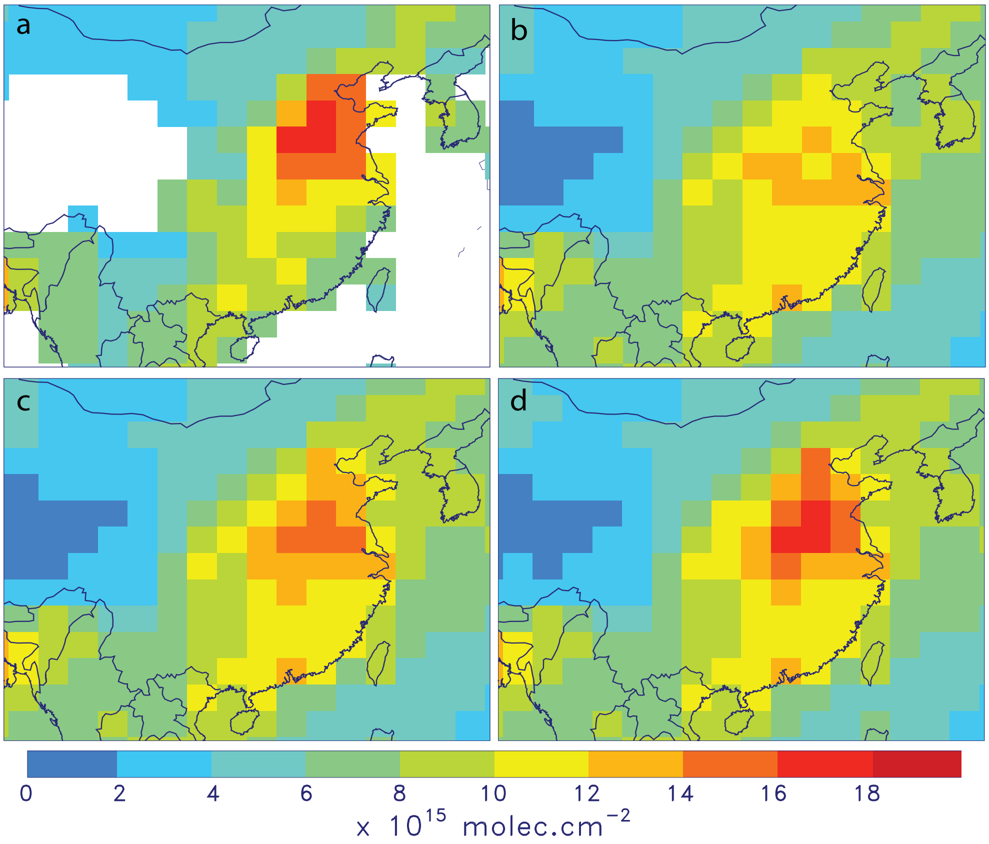

Figure S8: **June HCHO columns averaged over 2007-2012 (in 10^{15} molec.cm^{-2}).** **a,** Observed by GOME-2. A priori model HCHO column simulated **b**, assuming no crop fire fluxes, **c,** using the a priori crop fire fluxes, and **d,** using a posteriori fire emissions derived from the optimisation constrained by OMI HCHO columns. The maps were generated with IDL version 8.2.3 software (http://www.exelisvis.com).

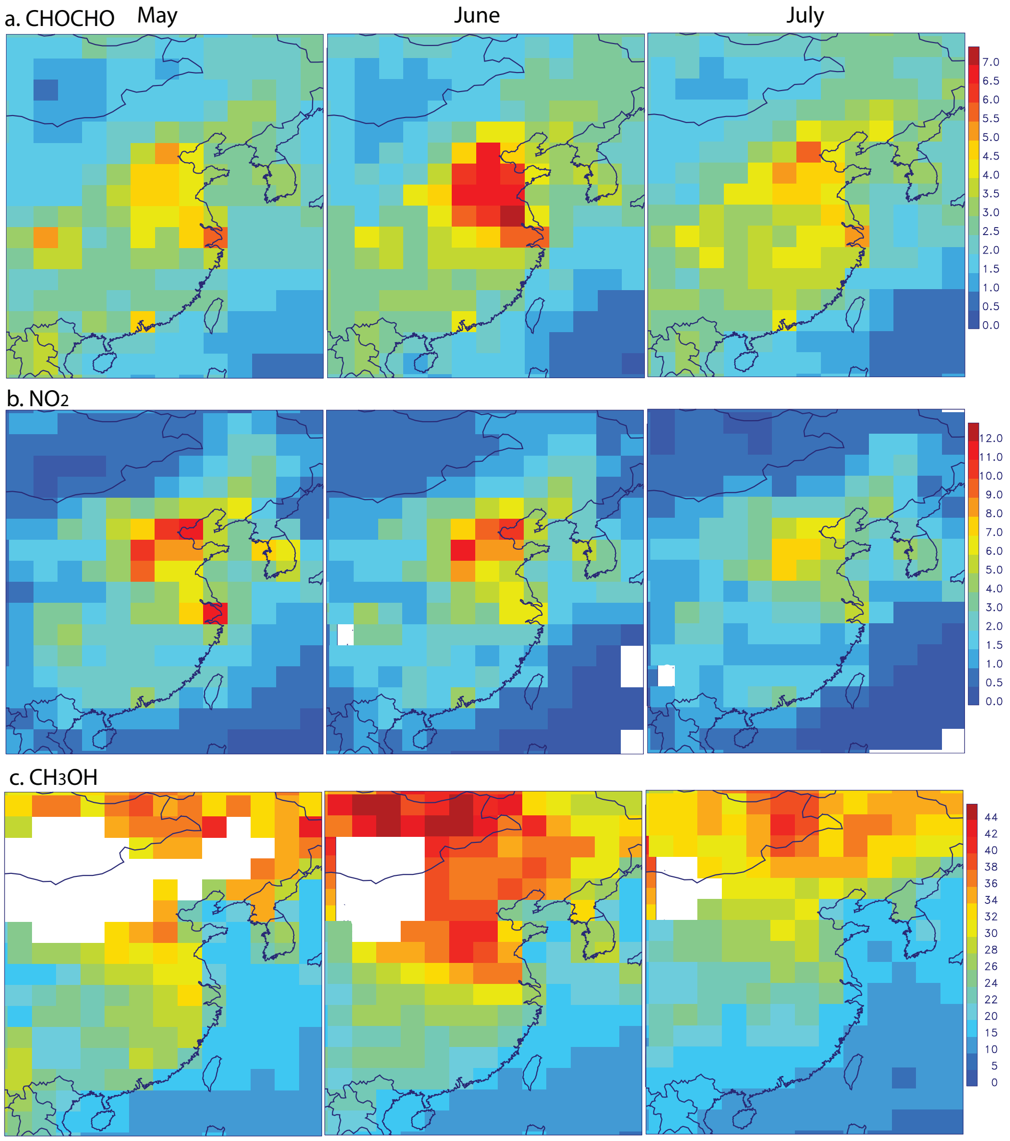

Figure S9: **Observed columns in May, June and July in China. a,** Glyoxal OMI columns averaged over 2005-2012 expressed in 10^{14} molec.cm^{-2}, **b,** NO_2 observed by OMI in 2012 expressed in 10^{15} molec.cm^{-2}, and **c,** methanol observed by IASI in 2009 expressed in 10^{15} molec.cm^{-2}. The maps were generated with IDL version 8.2.3 software (http://www.exelisvis.com).

References

[S1] De Smedt, I. *et al.* Diurnal, seasonal and long-term variations of global formaldehyde columns inferred from combined OMI and GOME-2 observations. *Atmos. Chem. Phys. Discuss.* **15**(8), 12241–12300 (2015).

[S2] Palmer, P. *et al.* Air mass factor formulation for spectroscopic measurements from satellites: Application to formaldehyde retrievals from the Global Ozone Monitoring Experiment. *J. Geophys. Res.***106**(D13), 14539–14550, doi:10.1029/2000JD900772 (2001).

[S3] Spurr, R. J. D. LIDORT and VLIDORT: Linearized pseudo-spherical scalar and vector discrete ordinate radiative transfer models for use in remote sensing retrieval problems. *Light Scattering Reviews*, edited by A. Kokhanovsky, pp. 229–271, Berlin (2008).

[S4] Kleipool, Q. L. *et al.* Earth surface reflectance climatology from 3 years of OMI data. *J. Geophys. Res.* **113**(D18), D18308, doi:10.1029/2008JD010290 (2008).

[S5] Martin, R. *et al.* An improved retrieval of tropospheric nitrogen dioxide from GOME. *J. Geophys. Res.***107**(D20), doi:10.1029/2001JD001027 (2002).

[S6] Wang, P. *et al.* FRESCO+: an improved O_2 A-band cloud retrieval algorithm for tropospheric trace gas retrievals. *Atmos. Chem. Phys. Discuss.* **8(3)**, 9697–9729, doi:10.5194/acpd-8-9697-2008 (2008).

[S7] Lerot, C. *et al.* Glyoxal vertical columns from GOME-2 backscattered light measurements and comparisons with a global model. *Atmos. Chem. Phys.* **10**, 12059–12072 (2010).

[S8] Alvarado, L. M. A. *et al.* An improved glyoxal retrieval from OMI measurements. *Atmos. Meas. Tech.* **7**(12), 4133–4150 (2014).

[S9] Miller, C. *et al.* Glyoxal retrieval from the Ozone Monitoring Instrument. *Atmos. Meas. Tech.* **7**(11), 3891–3907 (2014).

[S10] Clerbaux, C. *et al.* Monitoring of atmospheric composition using the thermal infrared IASI/METOP sounder. *Atmos. Chem. Phys.* **9**, 6041–6054 (2009).

[S11] Razavi, A. *et al.* Global distributions of methanol and formic acid retrieved for the first time from the IASI/Metop thermal infrared sounder. *Atmos. Chem. Phys.* **11**, 857–872 (2011).

[S12] Stavrakou, T. *et al.* First space-based derivation of the global atmospheric methanol emission fluxes. *Atmos. Chem. Phys.* **11**, 4873–4898 (2011).

[S13] Huang, X., Li, M., Li, J. & Song, Y. A high-resolution emission inventory of crop burning in fields in China based on MODIS Thermal Anomalies/Fire products. *Atmos. Environ.* **50**, 9–15 (2012).
